# Beyond CABG vs. PCI: Contemporary and Future Coronary Revascularisation from Historical Evolution to Artificial Intelligence, Robotics, and Hybrid Strategies

**DOI:** 10.3390/jcm15072681

**Published:** 2026-04-01

**Authors:** Justin Ren, Christopher M. Reid, Dion Stub, William Chan, Colin Royse, Jason E. Bloom, Garry W. Hamilton, Liam Munir, Gihwan Song, Daksh Tyagi, Joshua G. Kovoor, Aashray Gupta, Nilesh Srivastav, Alistair Royse

**Affiliations:** 1 Department of Surgery, The University of Melbourne, Melbourne, VIC 3052, Australia; 2 Department of Cardiothoracic Surgery, Royal Melbourne Hospital, Melbourne, VIC 3052, Australia; 3 Medical School, The University of Western Australia, Perth, WA 6009, Australia; 4 Population Health, Curtin University, Perth, WA 6102, Australia; 5 School of Public Health and Preventive Medicine, Monash University, Melbourne, VIC 3004, Australia; 6 Department of Cardiology, The Alfred Hospital, Melbourne, VIC 3004, Australia; 7 Department of Cardiology, Royal Melbourne Hospital, Melbourne, VIC 3004, Australia; 8 Baker Heart and Diabetes Institute, Melbourne, VIC 3004, Australia; 9 Department of Cardiology, Western Health, Melbourne, VIC 3021, Australia; 10 Department of Anaesthesia and Pain Management, Royal Melbourne Hospital, Melbourne, VIC 3004, Australia; 11 Outcomes Research, University of Texas Health, Houston, TX 77030, USA; 12 Department of Cardiology, Austin Health, Melbourne, VIC 3084, Australia; 13 Department of Medicine, University of Melbourne, Melbourne, VIC 3010, Australia; 14 School of Medicine and Dentistry, Griffith University, Gold Coast, QLD 4215, Australia; 15 School of Medicine, Deakin University, Melbourne, VIC 3125, Australia; 16 School of Medicine and Public Health, University of Newcastle, Newcastle, NSW 2308, Australia; 17 Future Health Systems, College of Health, Adelaide University, Adelaide, SA 5000, Australia; 18 School of Medicine, University of Adelaide, Adelaide, SA 5005, Australia; 19 Heart Vascular and Thoracic Institute, Cleveland Clinic Abu Dhabi, Abu Dhabi 112412, United Arab Emirates

**Keywords:** CABG, PCI, coronary intervention, coronary surgery, coronary stent

## Abstract

Coronary artery bypass grafting (CABG) and percutaneous coronary intervention (PCI) are the two dominant revascularisation strategies for obstructive coronary artery disease, yet their relative roles continue to shift because they address coronary pathophysiology differently with ever-evolving techniques. PCI has advanced through iterative improvements, including balloon angioplasty, bare-metal stents, and drug-eluting stents, with contemporary outcomes increasingly driven by procedural optimisation using intracoronary imaging and physiology-guided lesion selection rather than device category alone. CABG has progressed through perioperative management, improvements in operative safety, and, critically, conduit durability. Recognition of progressive saphenous vein graft failure has underpinned a conduit-optimisation era in which the left internal mammary artery to left anterior descending artery remains the gold standard. Further, broader arterial grafting (including radial artery use, multiple arterial grafting, and selected total-arterial strategies) has been increasingly applied, albeit with deliverability and competing-risk constraints highlighted in randomised evidence. This perspective review reframes the CABG versus PCI comparison not as a binary contest, but as a context-dependent assessment in which the relative value of each strategy depends on the specific technologies, techniques, and conduits available at the time of comparison. We summarise comparative effectiveness where evidence is most consistent and where it remains sensitive to anatomy, comorbidity, and endpoint definitions. In diabetes with multivessel disease, trial data favour CABG for long-term survival and clinical outcomes despite higher stroke risk. In left main disease, outcomes depend on lesion pattern and overall complexity, with trial-era stent technology and composite endpoint definitions influencing conclusions. In ischaemic left ventricular dysfunction, a long-term survival benefit is established for CABG added to medical therapy, while multi-vessel PCI has not demonstrated comparable prognostic modification in contemporary data. We then examine hybrid coronary revascularisation as territory-specific allocation, highlighting its physiological rationale, program dependence, and limited, adequately powered randomised evidence. Finally, we outline how artificial intelligence (AI) and robotics may accelerate a precision revascularisation paradigm by standardising lesion assessment, supporting procedural planning, improving procedural reproducibility, and enabling more patient-specific selection among PCI, contemporary CABG with optimised conduits, and hybrid pathways.

## 1. Introduction

Coronary artery bypass grafting (CABG) and percutaneous coronary intervention (PCI) remain the dominant revascularisation strategies for patients with obstructive coronary artery disease (CAD) [1], yet their comparative positioning continues to evolve. This is partly because the two therapies are biologically and anatomically distinct in how they modify coronary pathophysiology, and partly because both modalities are evolving. PCI has developed through stent design, intracoronary imaging, and physiology, whereas advances in CABG include conduit selection and handling, minimally invasive approaches, and perioperative care. Contemporary guidelines increasingly emphasise that optimal treatment selection requires multidisciplinary Heart Team decision-making and careful consideration of anatomy, comorbidities, procedural risk, and patient goals rather than a “one-size-fits-all” approach [2].

This perspective review is important because the CABG versus PCI debate is often presented as a binary contest, while contemporary practice is increasingly defined by context and execution. The same headline comparison can yield different conclusions depending on the technology used at the time, the conduit availability and planned strategy, and the coronary profiles being treated, which contributes to persistent uncertainty in daily decision-making despite a large evidence base. We therefore focus on three practical questions. First, how did PCI and CABG reach their current forms, and what do their historical challenges teach us about why outcome profiles diverge? Second, where does comparative effectiveness appear most consistent across major clinical populations, and where does it remain sensitive to anatomy, comorbidity, and endpoint definitions? Third, how are emerging hybrid pathways and new technologies reshaping the decision space?

## 2. Historical Evolution of Coronary Revascularisation

### 2.1. Evolution of Percutaneous Coronary Intervention (PCI)

**Figure 1:**
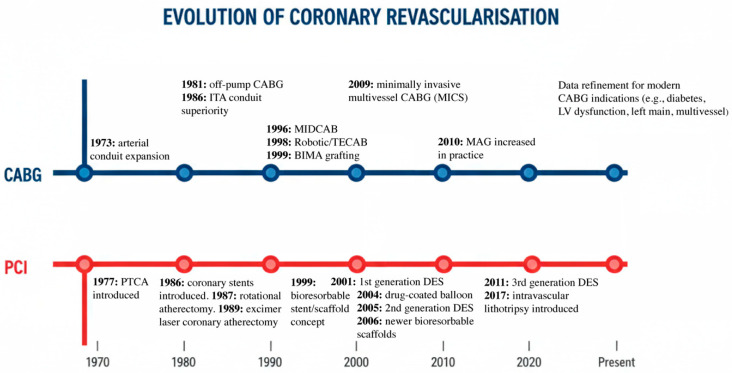
Evolution of coronary revascularisation: key milestones in CABG and PCI (1970–Present). CABG, coronary artery bypass grafting; PCI, percutaneous coronary intervention; ITA, internal thoracic artery; MAG, multiple arterial grafting; PTCA, percutaneous transluminal coronary angioplasty; MIDCAB, minimally invasive direct coronary artery bypass; TECAB, totally endoscopic coronary artery bypass; BIMA, bilateral internal mammary artery; MICS, minimally invasive cardiac surgery; DES, drug-eluting stent; LV, left ventricle.

Following the basic pattern of scientific evolution, PCI has advanced through an iterative cycle: a clinical limitation becomes a dominant mode of failure, the field builds a technical solution, and the solution introduces a new biology-driven complication that then defines the next era. The intellectual origin of coronary balloon angioplasty lies in the peripheral vascular work of Charles Dotter, who in 1964 performed the first intentional percutaneous transluminal angioplasty of a stenosed iliac artery using a coaxial catheter system. Andreas Grüntzig, inspired by Dotter’s concept, advanced it by developing a double-lumen balloon catheter that could dilate a stenosis without requiring progressively larger catheters, first applying it to femoral and renal arteries before performing his landmark first coronary balloon angioplasty on 16 September 1977 in Zürich (Figure 1) [3,4].

Early POBA delivered proof that coronary obstruction could be treated without open surgery, but also revealed the coronary artery was at times unforgiving. Acute vessel closure and early failure were common because balloon dilation was often associated with elastic recoil, flow-limiting coronary artery dissections, and acute thrombosis, while longer-term failure was dominated by constrictive remodelling (restenosis) due to proliferative healing responses. In that pre-stent era, restenosis after angioplasty was frequently reported in the 30–60% range, a magnitude that effectively set a “ceiling” on what balloon-only PCI could achieve at scale [5].

The introduction of bare-metal stents (BMSs) can be viewed as PCI’s first major philosophical shift: from *remodelling the vessel* to *implanting a permanent scaffold that restores vessel patency*. Stents directly addressed the POBA failure modes by preventing recoil and reducing constrictive remodelling, improving procedural predictability and lowering early complications—an enabling step that expanded PCI into more complex lesion subsets [6,7]. Yet the price of scaffolding was the creation of a new dominant late failure: in-stent restenosis (ISR) driven primarily by neointimal hyperplasia in response to metal-induced vascular injury. In clinical terms, ISR rates in the BMS era were commonly reported around 20–30% [8,9], re-framing restenosis from a “vessel problem” into a “device-vessel interaction problem”.

Drug-eluting stents (DESs) represented the next advancement, less a new scaffold than a strategy to pharmacologically reprogram the healing response at the site of intervention. By combining a stent platform with polymer-mediated delivery of antiproliferative agents, DESs substantially reduced neointimal hyperplasia and lowered restenosis rates compared with BMS, accelerating PCI’s expansion into multivessel disease and higher-risk anatomy. However, early-generation DESs also introduced concerns regarding late and very late stent thrombosis, particularly beyond 1 year [10], related to inadequate coverage of the stent struts by tissue, which prompted changes in device design, implantation technique, and antiplatelet strategies, all of which continue to be developed and critically appraised.

The contemporary PCI era is therefore less about a simple comparison of stent categories and more about how reliably PCI can be planned, executed, and optimised using current-generation drug-eluting stents, with the lifetime management of CAD in mind. Modern platforms have shifted toward thinner struts and more biocompatible polymer technologies, improving both deliverability and healing responses, but outcomes are increasingly determined by procedural quality rather than device choice alone [11]. In this context, optimisation strategies have become central to modern PCI practice. Intravascular imaging is increasingly used to confirm adequate stent expansion and apposition, detect edge complications, and clarify mechanisms of failure, with randomised evidence showing improved clinical outcomes when intra-coronary imaging is utilised to guide stent implantation compared with angiography guidance alone [12]. At the same time, physiology-guided lesion selection has reinforced the principle that angiographic severity variably predicts haemodynamic significance, and that targeting flow-limiting lesions can improve hard outcomes while reducing unnecessary stenting [13]. While PCI evolved through repeated refinements in device technology and procedural optimisation, CABG followed a parallel but distinct developmental pathway in which long-term success depended less on intracoronary device performance and more on conduit biology, graft durability, and operative strategy.

### 2.2. Evolution of Coronary Artery Bypass Grafting

The surgical aspiration to revascularise the ischaemic myocardium predates modern CABG. In the 1940s–1960s, the Vineberg procedure [14], direct implantation of a ligated internal mammary artery into a myocardial tunnel, represented an early attempt to restore perfusion without a direct anastomosis to the coronary artery. Although the operation achieved limited and inconsistent physiological benefit and was largely abandoned once direct aortocoronary bypass grafting became feasible, it introduced the internal mammary artery as a revascularisation conduit and can be regarded as the conceptual forerunner of modern CABG.

CABG has then progressed through two intertwined trajectories: improving the reliability of the operation (target selection, anastomotic technique, and perioperative safety) and improving the durability of the conduit that must function under arterial conditions for decades. The early modern CABG era was enabled by the technical feasibility of constructing reliable distal anastomoses and delivering rapid symptomatic relief, with saphenous vein grafts (SVGs) serving as a readily available conduit for multivessel revascularisation [15]. As follow-up matured, however, it became clear that long-term outcomes were not determined solely by the immediate technical success, but by whether the chosen conduit could adapt to coronary arterial haemodynamics and resist progressive atherosclerotic failure [16]. A defining inflexion point was the contemporary documentation of SVG failure as a staged process—from early thrombosis, followed by intimal hyperplasia, and later accelerated atherosclerosis—providing a coherent biological rationale for prioritising arterial conduits whenever feasible [17]. Put simply, arterial grafts tend to behave better because they are designed for the arterial environment: the internal mammary artery (IMA) has an endothelium resistant to atherogenesis and is characterised by more favourable vasoprotective signalling, including higher nitric oxide bioavailability and less pro-thrombotic, less proliferative vascular smooth muscle behaviour, which together mitigate intimal thickening over time [18]. In contrast, once a vein is implanted as a bypass conduit, it is abruptly exposed to arterial pressures and shear stress, which induces negative remodelling and represents a major mechanism of SVG attrition.

In a landmark Cleveland Clinic analysis [19], IMA use was associated with substantially better 10-year survival compared with vein-only grafting. As this evidence diffused into routine practice, the left internal mammary artery (LIMA) to the left anterior descending artery (LAD) became the procedural cornerstone of CABG and, in effect, its default first step whenever anatomically feasible, reflecting both the central prognostic importance of the LAD territory and the long-term reliability of the IMA conduit. Once LIMA-LAD became the gold standard, attention naturally turned to whether the same arterial advantage could be extended beyond the mammary artery to other conduits, most prominently the radial artery (RA). In an individual patient-level meta-analysis of randomised trials [20], RA grafting, compared with SVG, was associated with a lower risk of major adverse cardiac events (hazard ratio [HR], 0.67; 95% confidence interval [CI], 0.49–0.90; *p* = 0.01) and a substantially lower risk of graft occlusion (HR, 0.44; 95% CI, 0.28–0.70; *p* < 0.001). The finding was supported by our long-term retrospective angiographic data, in which RA grafts demonstrated patency and perfect patency broadly comparable to IMA grafts, with both arterial conduits clearly superior to SVGs. Among grafts that remained patent, almost all arterial conduits were also disease-free on angiography (perfectly patent), whereas a substantial proportion of patent SVGs showed angiographic disease, underscoring the tendency for progressive conduit atherosclerosis to disproportionately affect SVGs over time [21].

From that foundation, CABG entered a conduit-optimisation era in which multiple arterial grafting (MAG), defined by the use of more than one arterial conduit, and, in selected centres, total arterial revascularisation (TAR), where arterial conduits are used exclusively and SVGs are avoided altogether, have gained increasing interest. Large observational datasets have repeatedly reported reduced late adverse events associated with MAG and TAR compared with conventional single arterial grafting (SAG) [22,23,24]. At the same time, randomised evidence suggests that the benefit of expanding arterial grafting is contingent on real-world deliverability and competing risks. In the ART trial, the intention-to-treat comparison of bilateral versus single internal mammary artery grafting showed non-significant difference in 10-year mortality, illustrating how high rates of treatment-group crossover, concomitant radial artery use, and sternal wound complications can dilute or offset the expected long-term advantage of more extensive arterial grafting [25]. The frequent use of the radial artery in the single internal mammary artery group, now recognised to have durability and resistance to atherosclerosis comparable to internal mammary conduits [21], likely limited differentiation between strategies. In parallel, crossover between groups, often reflecting intraoperative surgical judgement, further diluted the intended treatment contrast. Finally, the higher incidence of sternal wound complications associated with bilateral internal mammary artery harvesting may have introduced competing early risks with potential downstream clinical consequences.

### 2.3. Lessons from Parallel Progress

A lesson from decades of parallel innovation is that CABG-PCI evidence [26] is best interpreted as comparisons between contemporaneous technology bundles, rather than definitive conclusions applicable across eras. Many landmark trials were conducted with PCI platforms that are now historical; for example, SYNTAX randomised patients to CABG versus PCI using paclitaxel-eluting stents, a first-generation drug-eluting platform, which limits direct extrapolation to contemporary thin-strut, biocompatible-polymer DES systems [27]. In contrast, the CABG arm has never been a single fixed intervention: completeness of revascularisation, arterial conduit utilisation beyond LIMA-to-LAD, and perioperative practice have varied widely, all of which can substantially influence long-term comparative outcomes. This moving-target phenomenon is a major reason why conclusions can diverge across trials, registries, and guideline updates, and why modern interpretation should explicitly state the stent generation, the use of imaging, and the conduit strategy being compared [28]. Crucially, technical advances do not invalidate earlier CABG and PCI comparisons, but they do reinforce the need to interpret them cautiously and within the clinical and technological context in which they were conducted.

The relative benefit of CABG versus PCI is influenced by the pattern of CAD, the patient’s risk profile, and, consequently, the capacity of the selected approach to deliver durable revascularisation, ventricular, and valvular function. CABG and PCI, therefore, should have distinct and complementary roles. PCI is well-suited to focal disease when durable percutaneous revascularisation is realistically achievable with a lower procedural risk, whereas CABG is favoured when disease is diffuse or complex and long-term durability is better supported by bypassing extensive atherosclerotic segments with durable conduits. The FAME 3 trial [29] provides a contemporary illustration of this principle. Despite the use of current-generation drug-eluting stents with physiology guidance, fractional flow reserve (FFR)-guided PCI did not meet noninferiority to CABG for the primary composite endpoint at one year in patients with three-vessel disease. Accordingly, optimal revascularisation strategy selection is best framed as a collaborative Heart Team decision that integrates coronary anatomy, comorbidity burden, procedural risk, institutional expertise, and patient goals rather than assuming a universal preference for either modality [28].

## 3. Comparative Effectiveness in Specific Clinical Populations

This historical perspective provides the necessary context for interpreting comparative effectiveness in contemporary practice, where the relative value of CABG and PCI becomes most clinically meaningful when examined within specific patient populations rather than as a universal comparison.

### 3.1. Diabetes Mellitus with Multivessel Disease

The FREEDOM trial [30] randomised patients with diabetes and multivessel coronary artery disease to CABG or PCI with DES and demonstrated superiority of CABG for the primary composite (*p* = 0.005) of all-cause mortality, myocardial infarction, or stroke. The benefit was driven mainly by fewer deaths and myocardial infarctions despite a higher stroke risk. An extended follow-up study [31] reported lower all-cause mortality with CABG (18.3%) than with PCI-DES patients (24.3%), suggesting there is a long-term prognostic advantage to CABG in the clinical context. Health status analyses have shown that CABG provides slightly improved intermediate-term quality of life and angina-related health status compared with PCI [32]. Guideline frameworks incorporate these data by generally favouring CABG in diabetics with extensive multivessel disease when surgical risk is acceptable, while preserving a role for PCI when surgical risk is prohibitive or when anatomy is favourable for durable percutaneous revascularisation [28]. Details are presented in Table 1.

### 3.2. Left Main Coronary Artery Disease

Left main coronary artery disease is one of the few settings where both strategies can be appropriate, but interpretation is highly dependent on lesion location, overall coronary complexity, and the era and definitions used in the trials. The EXCEL trial [33,34], which enrolled patients with left main disease of low to intermediate anatomic complexity, found no significant difference in the primary 5-year composite outcome between PCI with everolimus-eluting stents or CABG (absolute difference 2.8%; 95% CI, −0.9–6.5; *p* = 0.13), although all-cause mortality occurred more frequently after PCI than CABG (absolute difference 3.1%; 95% CI, 0.2–6.1). Interpretation of the EXCEL trial has been subject to ongoing debate, particularly regarding the definition of periprocedural myocardial infarction and the reporting of late mortality outcomes, underscoring the importance of consistent endpoint definitions when comparing revascularisation strategies. The NOBLE trial [35] of 1201 patients reported major adverse cardiac or cerebrovascular events (MACCE), a composite of all-cause mortality, non-procedural myocardial infarction, repeat revascularisation, and stroke. PCI was associated with significantly increased risk of MACCE (HR, 1.58; 95% CI, 1.24–2.01; *p* = 0.0002) at 5-year follow-ups, with similar all-cause mortality (HR, 1.08; 95% CI, 0.74–1.59; *p* = 0.68) but higher nonprocedural myocardial infarction (HR, 2.99; 95% CI, 1.66–5.39; *p* = 0.0002) and repeat revascularisation (HR, 1.73; 95% CI, 1.25–2.40; *p* = 0.0009) after PCI when compared to CABG. The PRECOMBAT trial [36], despite smaller cohorts, provides a longer horizon context. At 10 years, PCI with sirolimus-eluting stents and CABG were not significantly different for MACCE (HR, 1.25; 95% CI, 0.93–1.69). The observed inconsistency across trials reflects primarily the differences in the enrolled left main disease patterns and the stent technology used at the time of randomisation.

### 3.3. Reduced Left Ventricular Function

Reduced left ventricular function is an important subgroup, but there is no large, randomised trial that directly assigns patients with severe ischaemic cardiomyopathy to CABG versus multivessel PCI. Instead, the most informative evidence comes from parallel randomised programmes in broadly comparable phenotypes. At a median follow-up duration of 9.8 years, the STICHES trial [37] showed that CABG added to guideline-directed medical therapy improved long-term survival (HR, 0.84; 95% CI, 0.73–0.97; *p* = 0.02) compared with medical therapy alone in patients with ischaemic left ventricular (LV) dysfunction. In contrast, the REVIVED-BCIS2 trial [38] found that multivessel PCI added to optimal medical therapy did not reduce all-cause death or heart failure hospitalisation in patients with severe ischaemic LV dysfunction. A large Australian multicentre propensity analysis [39] between CABG versus PCI found that patients with severe ischaemic cardiomyopathy undergoing revascularisation by CABG rather than PCI showed improved long-term survival. Interpreted together, these studies support CABG as the revascularisation strategy with established prognostic benefit in appropriately selected patients with ischaemic cardiomyopathy when operative risk is acceptable, while highlighting that routine multivessel PCI has not yet demonstrated a comparable disease-modifying effect in this population. However, the lack of direct randomised comparative data would require cautious interpretation of these data.

### 3.4. Impaired Kidney Function

Patients with chronic kidney disease (CKD) represent a high-risk population in whom direct randomised comparisons of CABG versus PCI remain limited, as these patients are consistently underrepresented in major trials. Available evidence is largely observational and heterogeneous. Some studies suggest that CABG may be associated with more favourable long-term outcomes, including lower rates of myocardial infarction and repeat revascularisation, particularly in advanced CKD or dialysis-dependent populations, although these findings are not definitive [40]. The balance between improved durability of revascularisation and higher early procedural risk, including acute kidney injury and perioperative complications, further complicates decision-making. Overall, the current evidence base remains insufficient to draw firm conclusions, highlighting the need for dedicated studies in this population [41].

## 4. Hybrid Coronary Revascularisation

At the same time, the field has begun to move beyond strictly binary choices. Hybrid coronary revascularisation (HCR) can be conceptualised as a strategic integration of the most durable and prognostically important component of CABG with the procedural flexibility of PCI, thereby aiming to capture the complementary benefits of both approaches while mitigating their respective limitations. HCR is consistently described as a minimally invasive, sternal-sparing (or robotic/endoscopic) LIMA-to-LAD bypass paired with PCI using DES for secondary non-LAD targets. The intention is to preserve the long-term reliability of the mammary LAD graft while avoiding morbidity associated with sternotomy and cardiopulmonary bypass [42,43].

The rationale is also pragmatic. Despite guideline endorsement of multi-arterial grafting [28], SVGs remain the most common conduits used worldwide for non-LAD bypass targets [44], yet accumulating evidence underscores their limited durability, with failure rates of approximately 20% at 1 year and 70% by 15 years [45]. By contrast, contemporary DES may offer more durable outcomes than vein grafts for suitable non-LAD lesions [33,46]. HCR therefore seeks to match each coronary territory to the strategy most likely to provide sustained long-term performance, using LIMA-LAD grafting for the LAD and DES-based PCI for non-LAD disease when PCI is technically feasible and biologically well-suited. The underlying belief is that the LIMA-LAD is superior to any other grafting strategy, even with other arterial conduits. Notably, guideline recommendations remain cautious [47], with HCR assigned a Class IIb indication, reflecting the limited randomised evidence and the dependence on institutional expertise.

The dominant configuration is therefore LIMA-to-LAD first, followed by PCI to circumflex and right coronary territories, most commonly delivered as a staged CABG-then-PCI strategy [48]. This sequence is frequently favoured because it allows angiographic confirmation of LIMA-LAD patency at the time of PCI and simplifies perioperative antiplatelet planning, as dual antiplatelet therapy can be commenced after the surgical component rather than being carried into it. At the present time, the reasons why HCR remains a selective strategy in selected institutions are that it introduces coordination and logistical complexity, and it brings surgery into proximity with contrast exposure and antithrombotic therapy. Accordingly, its value depends heavily on careful case selection and a program capable of reliably delivering both components within an integrated coronary Heart Team model [49].

### Evidence, Current Positioning, and Practical Barriers

Whether this conceptual advantage of HCR would translate into clinical benefit remains an open question as the contemporary evidence base for HCR is dominated by small, randomised trials and larger observational comparisons, with meta-analytic pooling providing the most stable estimates (Table 2). In the most recent systematic review and meta-analysis [43] comparing HCR with conventional CABG for multivessel disease, there was no statistically significant difference in short-term mortality (odds ratio [OR], 1.50; 95% CI, 0.90–2.49; *p* = 0.11), postoperative stroke (OR, 1.36; 95% CI, 0.87–2.13; *p* = 0.16), or postoperative renal failure (OR, 0.71; 95% CI, 0.43–1.16; *p* = 0.14), whereas HCR was associated with fewer red cell transfusions (OR, 0.34; 95% CI, 0.22–0.54; *p* < 0.001) and shorter ICU stay (mean difference, −15.5 h; *p* < 0.001). This same meta-analysis reported no significant differences in midterm survival (OR, 0.86; 95% CI, 0.62 to 1.21; *p* = 0.39) or midterm major adverse cardiac events (MACE) (OR, 0.82; 95% CI, 0.55 to 1.23; *p* = 0.34). Randomised data remain limited and emphasise that outcomes are pathway dependent. In MERGING, reported by Esteves and colleagues [48], a pilot randomised trial enrolling patients with complex triple vessel disease, the primary 2-year composite of death, myocardial infarction, stroke, or repeat revascularisation occurred in 19.3% after HCR versus 5.9% after CABG, with the difference not statistically significant. Comparisons against multivessel PCI are mainly observational but include time-to-event analyses. In the prospective multicentre Hybrid Pilot Study [50] comparing HCR (*n* = 200) with multivessel PCI with DES (*n* = 98) in hybrid eligible anatomy, the risk-adjusted MACCE rate was similar at 12 months (HR, 1.06; 95% CI, 0.67 to 1.70), with continued follow up showing non-significant difference through end of study (HR, 0.87; 95% CI, 0.56 to 1.36). Another meta-analysis by Van den Eynde et al. comparing HCR with multivessel PCI showed no significant differences at 30 days across major endpoints, but at latest follow up HCR was associated with lower myocardial infarction (OR, 0.40; 95% CI, 0.20 to 0.80; *p* = 0.010) and lower target vessel revascularisation (OR, 0.49; 95% CI, 0.37 to 0.64; *p* < 0.001), while the difference for MACCE did not reach statistical significance (OR, 0.46; 95% CI, 0.20 to 1.05; *p* = 0.061) [51].

Hybrid coronary revascularisation has a compelling physiological and procedural rationale, but its evidence base and real-world implementation remain limited. The strongest quantitative data available still come from meta-analyses of predominantly non-randomised comparisons, which generally show comparable short-term safety to conventional CABG with signals of lower perioperative resource use [43]. What has not yet been established is a definitive long-term clinical advantage, largely because adequately powered randomised trials have been difficult to execute at scale.

Current positioning is therefore best framed as selective, as an option to be considered in specific subsets of patients at experienced centres, limited not by conceptual validity but by reproducible delivery. The Hybrid Coronary Revascularisation Trial provides a concrete illustration of why the field has struggled to move from plausible to proven. It was terminated early after severe recruitment difficulties [52], reflecting uneven institutional capacity and confidence in minimally invasive direct coronary artery bypass (MIDCAB) surgery. This dependency is inherent to the strategy. HCR only meaningfully differs from conventional CABG when the surgical component is delivered via a sternal-sparing approach, such as MIDCAB or robotic LIMA-LAD. However, these techniques remain technically demanding and program-dependent, limiting standardisation and broader adoption. Coordination requirements add a second level of complexity. Successful HCR requires integrated scheduling and consistent antiplatelet and bleeding risk management across procedural stages, effectively making HCR a specialised Heart Team pathway rather than a stand-alone procedure [53].

## 5. The Future: AI, Robotics, and Precision Revascularisation

The most credible near-term future for coronary revascularisation is not a single disruptive device, but a shift in how PCI and CABG decisions are made and executed. Across recent PCI-focused artificial intelligence (AI) literature, the unifying direction is standardisation. AI is being positioned to reduce the parts of coronary revascularisation that currently depend on subjective interpretation, to make lesion assessment and procedural optimisation more reproducible, and to convert tacit operator expertise into measurable outputs that can be audited and improved. In this conceptual framework, “precision revascularisation” is less about predicting outcomes in the abstract and more about improving the fidelity of the pathway from coronary phenotype to strategy selection to procedural execution.

### 5.1. AI and Robotics in PCI

Fully autonomous or semi-autonomous PCI is unlikely to be broadly implemented in the near term, whereas a more realistic trajectory would likely be decision-support algorithms that assist operators with case planning, lesion selection, and intraprocedural optimisation [54]. This matters because the catheterisation laboratory is a high-variance environment in which interpretation, technique, and workflow influence outcomes as much as device choice, and AI is being developed primarily to reduce that variability rather than to replace operators. Current AI development in PCI can be grossly grouped into three major functions.

The first is pre-procedural assessment and planning, where AI standardises lesion evaluation and functional decision-making before intervention. In angiography, AI-based fully automated quantitative coronary angiography (AI-QCA) has been validated against manual QCA, with one multi-centre study [55] demonstrating lesion detection sensitivity of 93% compared with 69% for visual estimation (*p* < 0.001), and a single-centre study [56] of 1002 angiograms confirming comparable accuracy to manual QCA across major vessels. Most notably, the FLASH trial [57] randomised 400 patients to AI-QCA-assisted versus optical coherence tomography (OCT)-guided PCI and demonstrated non-inferiority for achieving optimal minimal stent area, with comparable procedural complications and six-month outcomes.

The second is intraprocedural guidance and optimisation, where AI augments real-time imaging to reduce technical error during device deployment. For intravascular ultrasound, deep learning segmentation has achieved correlation coefficients >0.99 for lumen and vessel area measurements against expert analysis, enabling automated identification of landing zones, plaque burden thresholds, and minimal lumen area to guide stent sizing and positioning [58]. For OCT, the DeepNeo algorithm, trained on 1148 images and validated against histopathology, predicts neointimal tissue characterisation with 75% accuracy, comparable to expert classification, while substantially reducing assessment time and enabling standardised evaluation of stent healing at follow-up [59]. The clinical value is not that AI outperforms the expert operator, but that it makes optimal imaging-guided PCI easier to deliver consistently, particularly in complex anatomy where small technical deviations in expansion or apposition might have long-term consequences.

The third is long-term prognostic modelling, where machine learning integrates comorbidities and functional status with procedural and imaging variables to refine risk stratification beyond conventional scoring systems [60,61,62].

In parallel, robotic-assisted PCI (R-PCI) has a practical value proposition, with studies demonstrating significant reductions in staff radiation exposure [63,64,65]. The broader significance is that R-PCI provides a stable and reproducible execution platform that can translate algorithm-guided planning into consistent device manipulation at the table. Rather than outperforming expert manual PCI in isolation, robotics may add value by improving procedural precision and standardising key steps, particularly in complex cases where fatigue and small technical deviations can accumulate. It also creates a plausible pathway toward supervised remote intervention, although safe implementation would depend on systems factors such as network reliability, governance, credentialing, and an appropriately trained on-site team rather than technology alone [66,67].

### 5.2. AI and Robotics in CABG

Similar to PCI, the current role of AI in CABG is augmentation rather than autonomy, with the highest yield applications in decision support for planning and surveillance rather than automated anastomosis. Recent surgical reviews describe AI systems that integrate clinical and imaging data to support patient selection, conduit and target planning, and prediction of outcomes, including graft patency, mortality, and postoperative complications, including patient-specific three-dimensional reconstruction and simulation of alternative grafting strategies [68,69]. An engineering study [70] provides the mechanistic rationale, linking haemodynamic disturbance, abnormal shear stress, compliance mismatch, and anastomotic geometry to endothelial dysfunction, intimal hyperplasia, and graft failure, which makes computational modelling and machine learning attractive for quantifying modifiable failure risk rather than relying on specialist judgement alone. Deep learning surrogates for 3D haemodynamic prediction now deliver results approximately 600 times faster than traditional computational fluid dynamics, with approximately 90% accuracy, making patient-specific simulation of alternative grafting configurations feasible for preoperative planning [71]. A recent model incorporating both graft flow dynamics and geometric configuration achieved an area-under-curve (AUC) of 0.76 for predicting restenosis risk, with computational fluid dynamics (CFD)-confirmed correlation between predicted high-risk grafts and adverse haemodynamic conditions [72]. Emerging approaches include models using duplex-flow signals to predict graft occlusion or high-grade stenosis, supporting a pathway toward more data-driven planning and follow-up as external validation and workflow integration mature.

Robotic and endoscopic CABG is being adopted primarily to reduce access trauma while preserving the established durability of surgical revascularisation. Existing evidence [73,74] describes robotic platforms, most commonly the da Vinci system, as enabling smaller incisions and enhanced dexterity, with consistent short-term benefits such as reduced postoperative pain, quicker ambulation, tremor correction, and lower infection rates in selected patients [75]. The trade-off is deliverability. Totally endoscopic approaches require advanced training, are associated with longer operative and anaesthesia times during programme development, and have substantial infrastructure and cost barriers, with adoption highly dependent on surgeon experience, team coordination, and institutional resources [76]. Importantly, available comparisons have not consistently demonstrated a long-term graft patency advantage over conventional CABG.

## 6. Conclusions

CABG and PCI should be viewed as mature, complementary, though still constantly evolving revascularisation strategies whose comparative value is inherently context dependent. Their historical trajectories show that improvements in outcomes occur through identifying dominant failure modes and systematically reducing them through better devices, better conduit strategy, and better procedural execution. CABG remains most compelling when long-term durability depends on bypassing diffuse or complex disease with arterial conduits, whereas PCI is most compelling when focal disease can be treated with high-quality physiological selection and optimised deployment while minimising upfront morbidity. Across the populations reviewed, the consistent message is that anatomy, comorbidity burden, and achievable completeness of revascularisation are the principal modifiers of benefit, which is why multidisciplinary Heart Team decision-making remains central rather than optional.

Looking forward, the most important shift may be conceptual rather than technical. The CABG versus PCI debate remains useful insofar as it continues to generate comparative data, define patient phenotypes, and expose where each strategy fails, but its clinical utility is increasingly in enabling the next step, strategy individualisation. Hybrid revascularisation provides a practical demonstration that the field is already moving away from binary choices toward territory-specific allocation, while AI and robotics are likely to accelerate this trend by standardising coronary assessment, supporting physiology-informed planning, improving procedural reproducibility, and making outcome prediction more patient-specific. The likely endpoint is a precision revascularisation paradigm in which PCI, CABG with contemporary conduit selection, or hybrid pathways are selected to match the patient’s coronary anatomy, clinical profile, and goals, supported by validated decision tools rather than by default preferences for any single modality.

## Figures and Tables

**Table 1:** Landmark trials comparing CABG against PCI.

Trial	Institution	Country	Population	PCI Type	CABG Type	Main Outcomes	Key Message
FREEDOM	Mount Sinai School of Medicine	International, 140 centres	Diabetes + multivessel CAD	PCI with drug-eluting stents (mainly sirolimus-eluting and paclitaxel-eluting stents)	Arterial revascularisation encouraged	- Primary composite (death/MI/stroke) at 5 y: 26.6% PCI vs. 18.7% CABG (*p* = 0.005). - MI (*p* < 0.001) and death (*p* = 0.049) favoured CABG. - Stroke: 2.4% PCI vs. 5.2% CABG (*p* = 0.03).	In diabetes with multivessel CAD, CABG reduces death and MI compared with PCI-DES, with an early stroke trade-off.
EXCEL	Columbia University Medical Center	International (17 countries; 126 sites)	Left main CAD, low/intermediate anatomic complexity (site SYNTAX ≤ 32)	Fluoropolymer-based cobalt–chromium everolimus-eluting stents	Arterial grafts strongly recommended per protocol	- Primary composite (death/stroke/MI) at 3 y: 15.4% PCI vs. 14.7% CABG; HR 1.00 (95% CI, 0.79–1.26); non-inferiority *p* = 0.02; superiority *p* = 0.98.	PCI with contemporary EES was non-inferior to CABG for 3-year death/stroke/MI.
NOBLE (updated 5-year)	Aarhus University Hospital	Total of 36 hospitals, 9 northern European countries	Left main CAD	First-generation drug-eluting stents, then newer generation umirolimus-eluting stent	LIMA–LAD recommended; other conduits per practice	- In total, 5 y MACCE: 28% PCI vs. 19% CABG; HR 1.58 (95% CI, 1.24–2.01); *p* = 0.0002. - All-cause mortality: HR 1.08 (0.74–1.59). - Non-procedural MI: HR 2.99 (1.66–5.39). - Repeat revascularisation: HR 1.73 (1.25–2.40).	PCI was associated with worse 5-year composite outcome than CABG, driven largely by non-procedural MI and repeat revascularisation, while mortality was similar.
PRECOMBAT (extended 10-year)	Asan Medical Center, University of Ulsan College of Medicine	Korea, 13 hospitals	Unprotected left main CAD	Sirolimus-eluting stents	LAD preferentially with internal thoracic artery	- In total, 10 y MACCE: 29.8% PCI vs. 24.7% CABG; HR 1.25 (95% CI, 0.93–1.69). - Death/MI/stroke: HR 1.00 (0.70–1.44). - All-cause mortality: HR 1.13 (0.75–1.70). - Ischaemia-driven TVR: 16.1% vs. 8.0%; HR 1.98 (1.21–3.21).	At 10 years, no statistically clear difference in MACCE, but PCI had substantially more repeat revascularisation.
STICHES	Duke Clinical Research Institute	International (22 countries, 99 sites)	Ischaemic cardiomyopathy, LVEF ≤ 35%	N/A	CABG + guideline-directed medical therapy	- All-cause mortality: HR 0.84 (95% CI, 0.73–0.97; *p* = 0.02). - Cardiovascular death: HR 0.79 (0.66–0.93; *p* = 0.006). - Death or hospitalisation: HR 0.72 (0.64–0.82; *p* < 0.001).	Long-term survival benefit of CABG added to guideline-directed medical therapy in reduced LVEF.
REVIVED-BCIS2	St Thomas’ Hospital, London	United Kingdom, 40 centres	LVEF ≤ 35%	PCI strategy (multivessel PCI as feasible) plus optimal medical therapy	N/A	- Primary outcome: death from any cause or HF hospitalisation 37.2% PCI vs. 38.0% OMT; HR 0.99; 95% CI 0.78–1.27; *p* = 0.96	In severe LV dysfunction with viability on OMT, adding PCI did not reduce death or HF hospitalisation.

CABG, coronary artery bypass grafting; PCI, percutaneous coronary intervention; CAD, coronary artery disease; DES, drug-eluting stent; PCI-DES, percutaneous coronary intervention with drug-eluting stents; EES, everolimus-eluting stent; SYNTAX, synergy between PCI with TAXUS and cardiac surgery score; LIMA, left internal mammary artery; LAD, left anterior descending artery; LVEF, left ventricular ejection fraction; MACCEs, major adverse cardiac and cerebrovascular events; MI, myocardial infarction; TVR, target vessel revascularisation; HF, heart failure; OMT, optimal medical therapy; HR, hazard ratio; CI, confidence interval.

**Table 2:** Hybrid coronary revascularisation evidence summary with effect estimates.

Study (Year)	Institution	Design	Population	HCR	Comparator	Main Outcomes	Key Message
Systematic review and meta-analysis (Dixon et al., 2022) [43]	University of Bristol/Bristol Heart Institute	Systematic review and meta-analysis	Adults with multivessel CAD undergoing HCR vs. CABG	LIMA-LAD via mini-thoracotomy plus PCI to non-LAD vessels	Conventional CABG	- Short-term mortality OR 1.50 (95% CI 0.90–2.49; *p* = 0.11) - Stroke OR 1.36 (0.87–2.13; *p* = 0.16) - Renal failure OR 0.71 (0.43–1.16; *p* = 0.14) - Transfusion OR 0.34 (0.22–0.54; *p* < 0.001) - ICU stay mean difference −15.52 h (*p* < 0.001) - Mid-term survival OR 0.86 (0.62–1.21; *p* = 0.39) - Mid-term MACE OR 0.82 (0.55–1.23; *p* = 0.34)	HCR has similar short- and mid-term safety/outcomes to CABG in selected multivessel cohorts, with consistent signals for faster recovery (less transfusion, shorter ICU stay)
MERGING trial (Esteves et al., 2021) [48]	Heart Institute (InCor), University of São Paulo	Pilot randomised, open-label (2:1)	Complex triple-vessel disease, SYNTAX ≥ 22	Two-step HCR: off-pump LIMA-LAD via mini-thoracotomy, then PCI 48–72 h later with 2nd-gen DES (Promus Element everolimus)	Conventional CABG (LIMA–LAD strongly advised)	- Primary composite (death/MI/stroke/unplanned revasc) at 2 y: 19.3% HCR vs. 5.9% CABG (*p* = non-significant)	In complex 3-vessel disease, staged HCR was feasible but showed higher event rates than CABG over 2 years
Hybrid Revascularisation Observational Study (Puskas et al., 2016) [50]	Mount Sinai	Prospective multicentre observational; propensity adjustment	Hybrid-eligible multivessel CAD	Surgical LAD revascularisation (LITA/LIMA–LAD) plus PCI to ≥1 non-LAD target; staged completion within 6 weeks; DES at operator discretion	Multivessel PCI with DES	- MACCE at 12 months: adjusted HR 1.063 (95% CI 0.666–1.697) - MACCE through end of study: adjusted HR 0.868 (95% CI 0.556–1.355)	Risk-adjusted MACCE was similar between HCR and multivessel PCI over 12 months
Systematic review and meta-analysis (Van den Eynde et al., 2021) [51]	KU Leuven/University Hospitals Leuven	Systematic review and meta-analysis	Multivessel CAD undergoing HCR vs. multivessel PCI	HCR (typically LITA/LIMA–LAD + PCI to non-LAD targets)	Multivessel PCI	- MI OR 0.40 (95% CI 0.20–0.80; *p* = 0.010) - TVR OR 0.49 (0.37–0.64; *p* < 0.001) - MACCE OR 0.46 (0.20–1.05; *p* = 0.061) - 30-day: no significant differences in major endpoints	Compared with multivessel PCI, HCR shows similar early outcomes and lower MI/TVR at latest follow-up in pooled data

HCR, hybrid coronary revascularisation; CABG, coronary artery bypass grafting; PCI, percutaneous coronary intervention; CAD, coronary artery disease; LIMA, left internal mammary artery; LITA, left internal thoracic artery; LAD, left anterior descending artery; SYNTAX, synergy between PCI with TAXUS and cardiac surgery score; DES, drug-eluting stent; ICU, intensive care unit; MACE, major adverse cardiac events; MACCEs, major adverse cardiac and cerebrovascular events; MI, myocardial infarction; TVR, target vessel revascularisation; OR, odds ratio; HR, hazard ratio; CI, confidence interval.

## Data Availability

No new data were created or analysed in this study.
